# Src acts as the target of matrine to inhibit the proliferation of cancer cells by regulating phosphorylation signaling pathways

**DOI:** 10.1038/s41419-021-04221-6

**Published:** 2021-10-12

**Authors:** Xi Zhang, Hui Xu, Xiaoyang Bi, Guoqing Hou, Andong Liu, Youyun Zhao, Guoping Wang, Xuan Cao

**Affiliations:** 1 Department of Medical Genetics, School of Basic Medicine, Tongji Medical College, Huazhong University of Science and Technology, Wuhan, 430030 China; 2 School of Pharmacy, Xinxiang Medical University, Xinxiang, 453003 Henan China; 3 Department of Pathology, School of Basic Medicine, Tongji Medical College, Huazhong University of Science and Technology, Wuhan, 430030 China; 4 Department of Clinical Laboratory, Hubei Provincial Hospital of Traditional Chinese Medicine, Wuhan, 430073 China; 5 Institute of Pathology, Tongji Hospital, Tongji Medical College, Huazhong University of Science and Technology, Wuhan, 430030 China

**Keywords:** Targeted therapies, Cell growth

## Abstract

Studies have shown that matrine has antitumor activity against many types of cancers. However, the direct target in cancer cells of its anticancer effect has not been identified. The purpose of this study was to find the molecular target of matrine to inhibit the proliferation of cancer cells and explore its mechanism of action. Herein we showed that matrine inhibited the proliferation of cancer in vitro and in vivo. Pull-down assay with matrine-amino coupling resins and liquid chromatography-mass spectrometry/mass spectrometry (LC-MS/MS) identified Src as the target of matrine. Cellular thermal shift assay (CETSA) and drug affinity responsive target stability (DARTS) provided solid evidences that matrine directly bound to Src. Bioinformatics prediction and pull-down experiment demonstrated that Src kinase domain was required for its interaction with matrine and Ala392 in the kinase domain participated in matrine–Src interaction. Intriguingly, matrine was proven to inhibit Src kinase activity in a non-ATP-competitive manner by blocking the autophosphorylation of Tyr419 in Src kinase domain. Matrine down-regulated the phosphorylation levels of MAPK/ERK, JAK2/STAT3, and PI3K/Akt signaling pathways via targeting Src. Collectively, matrine targeted Src, inhibited its kinase activity, and down-regulated its downstream MAPK/ERK, JAK2/STAT3, and PI3K/Akt phosphorylation signaling pathways to inhibit the proliferation of cancer cells.

## Introduction

Cancer is the most common disease and remains a great threat to human health worldwide. As the conventional remedial methods, chemotherapy, and radiotherapy showed strong side effects which limit their clinical application [1]. Based on the advantages of specifically killing cancer cells without significant toxicity to normal tissues or cells, molecular targeted therapy has achieved good therapeutic effects in cancer patients during the past decades [2]. Molecular targeted therapy drugs can target specific receptors or kinases and preferentially block the signal pathways, thus inhibiting the cell proliferation and eventually leading to cell death [3–5].

Protein phosphorylation is one of the most common and important post-translational modifications (PTMs), which serves as a mechanism to regulate the activity of enzyme or protein–protein interaction in cellular signal transduction [6, 7]. Protein kinases may catalyze the transfer of a phosphoryl group from ATP to tyrosine, serine, or threonine residue of its substrate proteins. Overexpression or aberrant activation of protein kinases can result in a variety of diseases, such as cancer. Thus, protein kinases are the popular targets for the treatment of cancer [8].

With the virtues of low side toxicity and therapeutic selectivity, some novel natural compounds from medicinal plants have been developed to prevent or treat the occurrence and metastasis of cancer, which presents a compelling tendency for cancer therapy. Matrine is one of the alkaloids isolated from the traditional Chinese medicine *Sophora flavescens* Aiton, exhibiting a wide spectrum of pharmacological activities [9]. Matrine has been widely studied in many kinds of cancers, including lung cancer, breast cancer, liver cancer, gastric carcinoma, pancreatic cancer, ovarian cancer, and leukemia. Intensive studies confirmed the antitumor mechanisms of matrine, including regulation of phosphorylation of proliferation- and apoptosis-related cell signaling pathways [10, 11]. It has been reported that matrine inhibits cell proliferation of human rhabdomyosarcoma cells via inactivation of the ERK signaling [12]. Matrine can reduce the phosphorylation levels of Janus kinase 2 (JAK2) and signal transducer and activator of transcription 3 (STAT3) and inhibit STAT3-dependent transcriptional activity in human cholangiocarcinoma cells [13], and inhibit the proliferation and invasion of bladder cancer cells through the PI3K/AKT signaling pathway [14].

Although the antitumor effects of matrine have been extensively studied, its direct targets and the precise molecular mechanisms that play the anticancer role remain to be elusive. In the present study, we designed and synthesized a novel matrine-amino coupling resin (MA beads) to explore the direct targets of matrine and related cell signaling pathways, and revealed the molecular mechanisms of matrine inhibiting the proliferation of cancer cells, providing strong shoring of foundation for the development of active ingredients of traditional Chinese medicine as novel targeted therapy drugs against cancer.

## Results

### Matrine inhibits the proliferation of cancer in vitro and in vivo

**Fig. 1:**
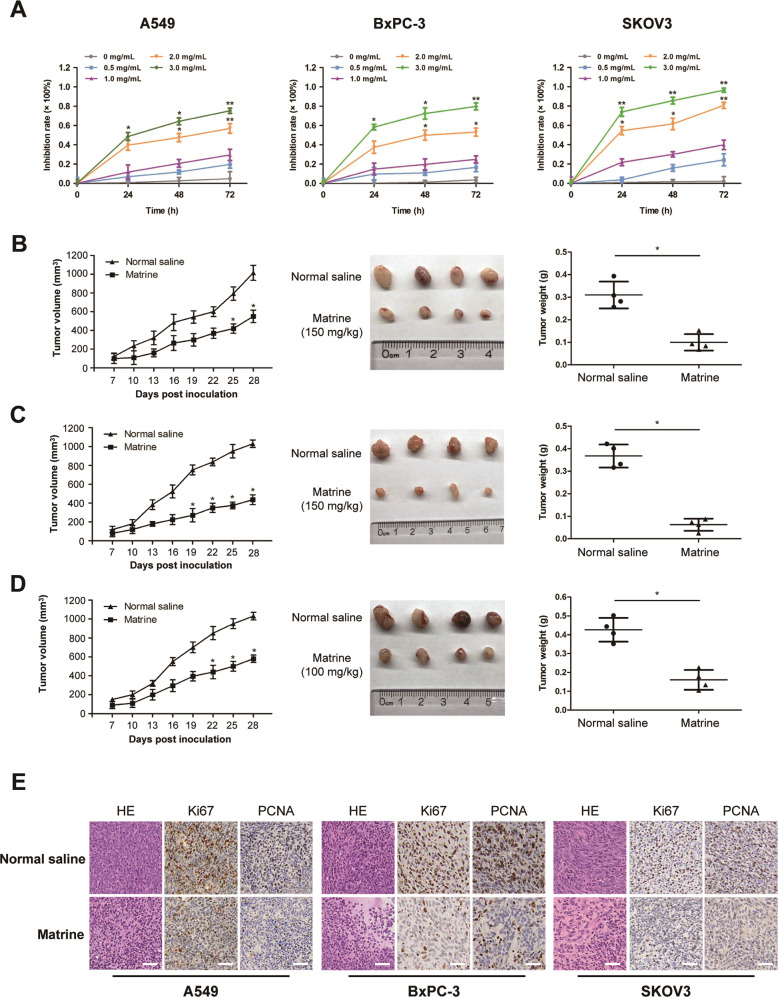
Matrine inhibited the proliferation of cancer cells in vitro and in vivo. **A** Human non-small cell lung cancer cell A549, human pancreatic cancer cell BxPC-3, and human ovarian cancer cell SKOV3 were treated with matrine at the concentrations of 0, 0.5, 1.0, 2.0, and 3.0 mg/mL for 0, 24, 48, and 72 h. MTT assay was conducted to examine the effect of matrine on the proliferation of cancer cells. Data are presented as mean ± SD (*P < 0.05, **P < 0.01). **B–D** Xenograft models were established by subcutaneous injection of A549 (**B**), BxPC-3 (**C**), and SKOV3 (**D**) cells in BALB/c nude mice, respectively. Tumor volume, size, and weight in matrine-treated mice were significantly smaller than those in normal saline-treated mice (150 mg/kg in A549 and BxPC-3 cells, 100 mg/kg in SKOV3 cells, i.p.). Data are presented as mean ± SD (*n* = 4, *P < 0.05). **E** The representative histological examinations of dissected tumors of mice inoculated with A549, BxPC-3, and SKOV3 cells after matrine or normal saline treatment. Scale bar: 50 μm. Results are representative of three experiments.

The effects of matrine on the proliferation of cancer cells, including human colon cancer cell HT-29, human breast cancer cell MCF7, human non-small cell lung cancer cell A549, human pancreatic cancer cell BxPC-3, human ovarian cancer cell SKOV3, and human cervical cancer cell HeLa, were determined by MTT assay. Each cell line was treated with matrine at concentration of 0, 0.5, 1.0, 2.0, or 3.0 mg/mL for 0, 24, 48, or 72 h. The results showed that matrine inhibited the proliferation of cancer cells in a dose- and time-dependent manner (Fig. 1A and Supplementary Fig. S1A). The optimal concentration of matrine was 2.5, 2.5, 3.0, 3.0, 2.0, and 2.5 mg/mL in HT-29, MCF7, A549, BxPC-3, SKOV3, and HeLa cells, respectively (Supplementary Fig. S1B). To further determine the anticancer effects of matrine in vivo, we injected subcutaneously cancer cells, including A549, BxPC-3, or SKOV3 cells into BALB/c nude mice, respectively. After inoculation, the mice were treated with an intraperitoneal injection of matrine or normal saline thrice a week. The results demonstrated that tumor volume, size, and weight of mice in the matrine group were found to be much smaller than those in the normal saline group (Fig. 1B–D). Hematoxylin and eosin (H&E) staining indicated that matrine caused necrosis lesions in tumor tissues of mice inoculated with three cancer cells. Immunohistochemistry (IHC) analysis showed that treatment with matrine resulted in a remarkably smaller proportion of proliferation marker proteins Ki-67 and PCNA positive cancer cells in tumors compared with the control group (Fig. 1E). Moreover, histopathological analysis of heart, liver, spleen, lung, and kidney of matrine-treated mice showed no obviously change compared to the control group (Supplementary Fig. S2), which suggested that matrine could be a safe agent to cancer in vivo. All these data demonstrated that matrine inhibited the proliferation of cancer in vitro and in vivo without obvious toxicity.

### Src is the potential target of matrine

**Fig. 2:**
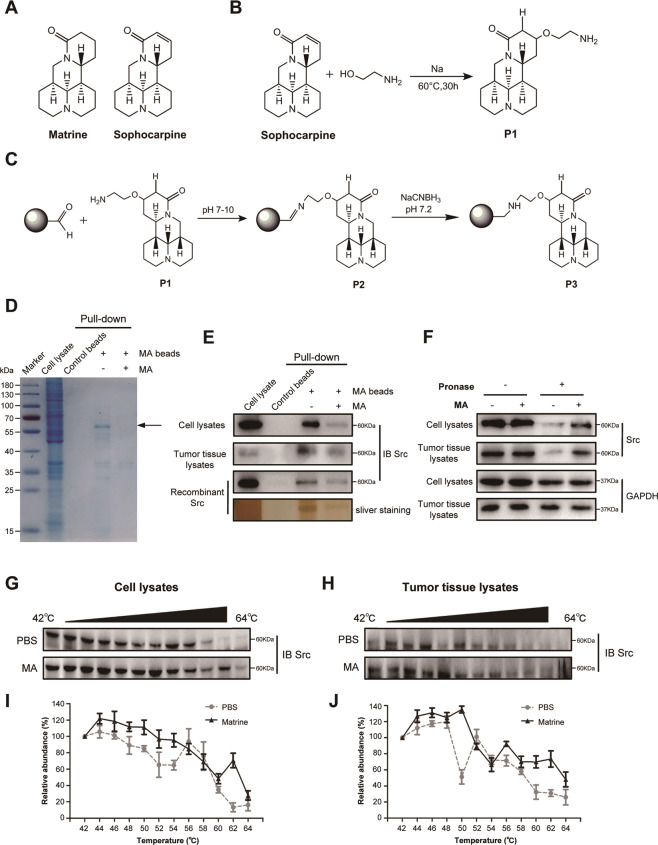
Matrine targeted Src protein. **A** Chemical structure of matrine and sophocarpine. **B** The synthesis scheme of 13α-(2-amino) ethoxymatrine (P1). **C** Synthesis of matrine-amino coupling resins (MA beads, P3). **D** SKOV3 cell lysates were incubated with control beads, MA beads, and MA beads in the presence of matrine (2.0 mg/mL). Protein affinity pull-down assay was performed and the matrine-bound proteins were separated by SDS-PAGE. The gels were processed by Coomassie brilliant blue staining. **E** Pull-down assays were performed using cell lysates, tumor tissue lysates, and recombinant protein GST-Src. The cell lysates, tumor tissue lysates, and recombinant proteins were incubated with control beads, MA beads, or MA beads in the presence of matrine (2.0 mg/mL). The mixtures were blotted for Src and sliver staining. **F** DARTS results for pronase-digested cell lysates and tumor tissues. Cell lysates and tumor tissue lysates were prepared with matrine or PBS for 1 h, followed by digestion with pronase. Western blotting showed protection of the target protein Src, whereas digestion of the non-target proteins GAPDH is unchanged by matrine. **G**, **H** CETSA was performed in cell lysates (**G**) and tumor tissue lysates (**F**) treated with matrine or PBS. The stabilization of matrine on Src protein was evaluated by immunoblot. **I**, **J** CETSA curves in cell lysates (**I**) and tumor tissue lysates (**J**) treated with matrine or PBS. Data are presented as mean ± SD, *n* = 3 independent experiments.

Above data showed that matrine is a potent inhibitor of cancer growth in vitro and in vivo. Then, we sought to explore the potential targets of matrine that mediate its growth inhibitory capability. Matrine and Sophocarpine are alkaloids extracted from the traditional Chinese medicine *Sophora flavescens* Aiton (Fig. 2A). To explore the potential targets of matrine, we prepared chemical probes for affinity purification. Sophocarpine, the structural analog of matrine, was used to synthesize 13α-(2-amino) ethoxymatrine (P1) (Fig. 2B). High-resolution mass spectrum (HRMS) of P1 was gathered on a Bruker MicroTOF-Q III LC-MS instrument operating in electrospray ionization (ESI) (Supplementary Fig. S3A). As shown in Fig. 2C, matrine–amino coupling resins (MA beads, P3) were made by coupling reaction of 13α-(2-amino) ethoxymatrine with aminolink coupling resins, which were used to pull down the cellular targets of matrine. In addition, competition assay with matrine–amino coupling resins was carried out by adding matrine during coupling reaction. The proteins retained by MA beads were separated by sodium dodecyl sulfate-polyacrylamide gel electrophoresis (SDS-PAGE) and visualized by Coomassie brilliant blue staining. The results showed that one obvious protein band could be observed between 55 and 70 kDa in the group with MA beads but not in group with control beads. Moreover, this protein band was found much weaker with an excess amount of matrine for competition (Fig. 2D). The band in the gel was excised and identified by liquid chromatography-mass spectrometry/mass spectrometry (LC-MS/MS). Mass spectrometric analysis verified this protein as Src protein (Supplementary Table S1).

To further confirm that Src is the direct target protein of matrine, affinity pull-down assay was performed with matrine-amino coupling resins (MA beads) and matrine. As shown in Fig. 2E, Src protein could be precipitated by MA beads, and increasing amount of matrine competitively inhibited the binding between Src and MA beads in cell lysates and tumor tissue lysates. Meanwhile, recombinant protein GST-Src could also be precipitated by MA beads, and competitively inhibited by addition of matrine in immunoblot and sliver staining assays. Furthermore, drug affinity responsive target stability (DARTS) results showed that the presence of matrine partially prevents pronase-mediated digestion of Src. Src protein is mostly digested in the absence of matrine, whereas most of them is undigested in the presence of matrine in cell lysates and tumor tissue lysates. GAPDH was used as a negative control, and it was not affected by matrine (Fig. 2F). As ligand may contribute to the stabilization of its target proteins, cellular thermal shift assay (CETSA) was carried out to confirm the interaction between Src and matrine in vitro. The thermal stability of Src protein in cell lysates (Fig. 2G) and tumor tissue lysates (Fig. 2H) was tested by western blot at the temperature range of 42–64 °C after exposing to matrine or PBS. The results showed that Src was still obviously detectable at the temperature of 60–64 °C in the group exposed to matrine, but not in the control group. Matrine treatment efficiently protected Src protein from temperature-dependent degradation. The significant shift in melting temperatures of Src protein and hence stabilization upon addition of matrine indicated that matrine directly bound to Src protein (Fig. 2I, J). Overall, these results suggested that Src was the direct target of matrine in cancer cells.

### Src kinase domain is required for its interaction with matrine

**Fig. 3:**
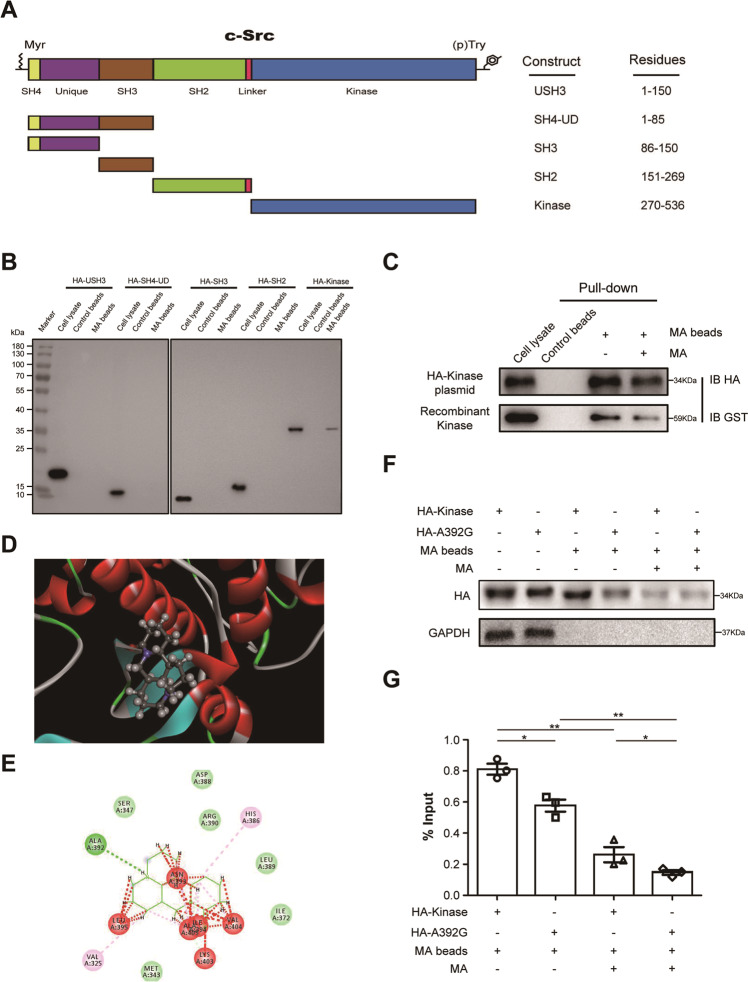
Src kinase domain was required for its interaction with matrine. **A** Schematic of constructs with different functional domains of Src. USH3: 1–150 aa, SH4-UD: 1–85 aa, SH3: 86–150 aa, SH2: 151–269 aa, Kinase: 270–536 aa. **B** HA-tagged deletion constructs of Src, including HA-USH3, HA-SH4-UD, HA-SH3, HA-SH2, and HA-Kinase, were transfected into HEK-293 cells, respectively. Cell lysates were harvested and incubated with MA beads or control beads and blotted by anti-HA. **C** Constructs containing Src kinase domain were overexpressed in HEK-293 cells and expressed in BL21 cells, respectively. Competition assay results showed that cell lysates transfected with HA-Kinase plasmid and recombinant protein were pulled down by MA beads, which were reduced by adding an excess amount of MA. **D** Molecular docking diagram of matrine bound to Src kinase domain (PDB: 1YOJ). Matrine depicted as the ball-and-stick model showing carbon (gray), hydrogen (white), oxygen (red), and nitrogen (blue) atoms. **E** Hydrogen bonds between matrine and Src kinase domain were identified and indicated by green line. Van der Waals forces between matrine and Src kinase domain were indicated by pale green circles. **F** HEK-293 cells were transfected with plasmids pcDNA3.1-HA-Kinase and pcDNA3.1-HA-Kinase-A392G, respectively. MA beads were added to cell lysates for pull-down assay to detect matrine binding. **G** Densitometric quantification data of matrine binding followed by pull-down assay. Data are presented as mean ± SD (*n* = 3, *P < 0.05, **P < 0.01).

From the N to C terminus, Src contains an SH4 domain, a unique domain, an SH3 domain, an SH2 domain, an SH2-kinase linker, a protein-tyrosine kinase domain, and a regulatory tail (Fig. 3A). Src segmented plasmids, including pcDNA3.1-HA-USH3, pcDNA3.1-HA-SH4-UD, pcDNA3.1-HA-SH3, pcDNA3.1-HA-SH2, and pcDNA3.1-HA-Kinase, were constructed and overexpressed in HEK-293 cells, respectively. The corresponding cell lysates were incubated with MA beads to determine matrine-binding region of Src, which then were dissociated from the beads by denaturation and were probed with anti-HA. Pull-down assay demonstrated that protein band precipitated by MA beads could be only observed in cell lysates transfected with plasmid pcDNA3.1-HA-Kinase. The results indicated that matrine bound directly to the Src kinase domain, and its ability to interact with other regions of Src was negligible (Fig. 3B). To confirm that matrine may definitely bind to the kinase domain of Src, binding competition assay was conducted using pcDNA3.1-HA-Kinase plasmid and GST-Kinase fusion protein. As shown in Fig. 3C, Kinase proteins were pulled down by MA beads, which were reduced by adding an excess amount of MA in cells transfected with plasmid pcDNA3.1-HA-Kinase and in GST-Kinase recombinant protein.

To dissect the critical amino acids involved in the interaction between Src and matrine, the molecular docking of matrine with Src kinase domain was predicted by Discovery Studio 4.5 software. Three-dimensional (3D) molecular dynamics simulation analysis revealed that matrine was embedded in the cleft of the Src kinase domain. Van der Waals forces were also formed between matrine and the residues of kinase domain, including Met343, Ser347, Ile 372, Asp388, Leu389, and Arg390. Conspicuously, a strong hydrogen-bonding interaction existed between matrine and the residue of Ala392 (Fig. 3D, E). Then, Ala392 in the kinase domain was mutated to Gly to confirm its involvement in matrine–Src interaction. Plasmid pcDNA3.1-HA-Kinase-A392G was constructed and transfected into HEK-293 cells as well as pcDNA3.1-HA-Kinase. Pull-down assay was performed to determine whether matrine binds to mutated kinase domain of Src in the transfected cells using MA beads. The results showed that matrine bound to wild-type HA-Kinase, while significantly reduced binding was observed for mutant Kinase-A392G. Moreover, the binding was also attenuated in cell lysates expressing mutant plasmid by addition of MA (Fig. 3F, G). These results suggested that Ala392 in the kinase domain exhibited an important role in matrine–Src interaction.

### Matrine is a non-ATP-competitive inhibitor of Src kinase

**Fig. 4:**
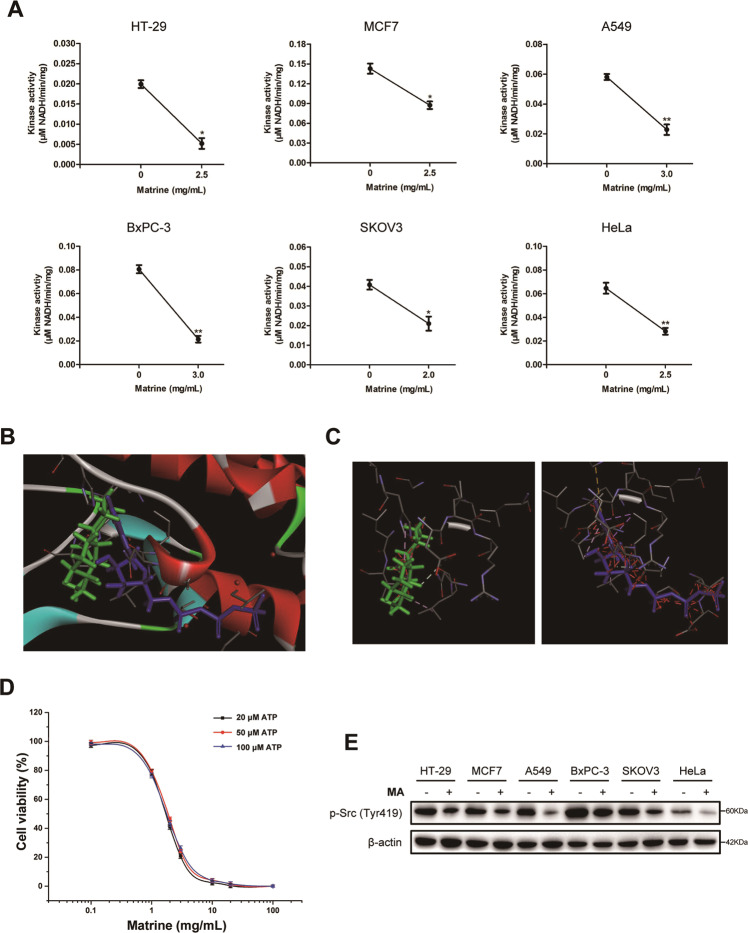
Matrine was a non-ATP-competitive inhibitor of Src kinase. **A** The effect of matrine on Src kinase activity in cancer cells, including HT-29, MCF7, A549, BxPC-3, SKOV3, and HeLa, were determined according to the instructions of Src kinase activity detection kit. Results are representative of three experiments (*P < 0.05, **P < 0.01). **B**, **C** Schematic diagram of matrine and ATP bound to Src kinase domain. Matrine (green) and ATP (bule) molecules were depicted as the ball-and-stick models. **D** Dose–response curves of matrine against Src kinase at various ATP concentrations. Data are the mean ± SD of three independent experiments. **E** HT-29, MCF7, A549, BxPC-3, SKOV3, and HeLa cells were exposed to matrine at its optimal concentrations for 24 h, respectively. The effects of matrine on the p-Src (Tyr419) were analyzed by western blot, respectively. β-Actin was used as a control.

The tyrosine kinase Src is activated in a large number of human malignancies and plays significant roles in the development of cancers. The biochemical and structural basis for the basal inhibition of Src protein tyrosine kinase activity was largely elucidated at present [15–17]. To investigate the effect of matrine on Src kinase activity, cells were treated with the optimal concentration of matrine, respectively. The results showed that after treated with matrine, the Src kinase activities in cancer cells, including HT-29, MCF7, A549, BxPC-3, SKOV3, and HeLa, were significantly decreased (Fig. 4A). ATP-competitive inhibitors have been developed and some are used in clinical research, whereas their inhibitory activities must be strong enough to compete with endogenous ATP [18, 19]. Moreover, a number of non-ATP-competitive inhibitors have also been reported for kinases [20]. Molecular docking results indicated that both matrine and ATP bound to Src kinase domain (Fig. 4B, C). To investigate the competitive interaction between matrine and ATP, we measured IC_50_ values of matrine at various ATP concentrations in SKOV3 cells. The dose–response curves of matrine at ATP concentrations of 25, 50, and 100 μM are indistinguishable. The corresponding IC_50_ values are 1.83 ± 0.57, 2.15 ± 0.34, and 2.05 ± 0.63 mg/mL. This verified that matrine is a non-ATP-competitive inhibitor of Src kinase domain (Fig. 4D).

It has been shown that protein tyrosine kinase could selectively catalyze phosphorylation of the tail tyrosine site in Src kinase domain [21]. Dephosphorylation of Y530, binding of SH2 and/or SH3 ligands, and phosphorylation of Y419 of the activation loop may activate the Src kinase [22, 23]. Cancer cells, including HT-29, MCF7, A549, BxPC-3, SKOV3, and HeLa, were exposed to matrine at its optimal concentrations for 24 h, respectively. The phosphorylation level of Src Y419 was detected by immunoblot. The results indicated that matrine obviously decreased the tyrosine 419 phosphorylation level of Src after matrine treatment in cancer cells (Fig. 4E). Considering that Src Ala392 is adjacent to Y419, we speculated that matrine might block the autophosphorylation of tyrosine 419, resulting in inactivation of Src kinase.

### Matrine inhibits phosphorylation signaling pathways in cancer cells

**Fig. 5:**
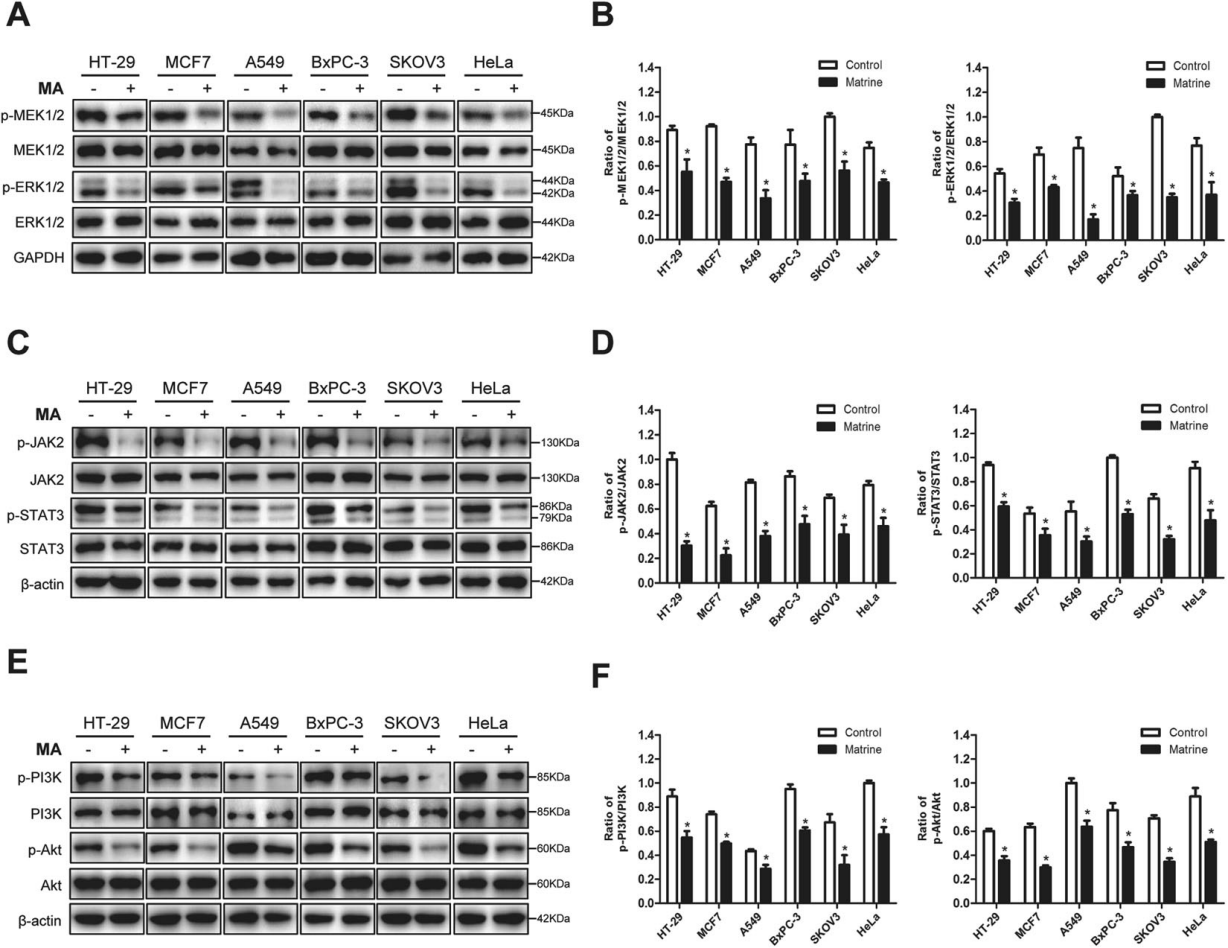
The phosphorylation signaling pathways were inhibited by matrine. **A** Cancer cells, including HT-29, MCF7, A549, BxPC-3, SKOV3, and HeLa, were treated with matrine at the optimal concentration for 24 h, respectively. The effects of matrine on the phosphorylation levels of MEK1/2 and ERK1/2 in cancer cells were detected by western blot. GAPDH was used as a loading control. **B** Histograms indicating the phosphorylation ratio of MEK1/2 and ERK1/2 in cancer cells. Data are presented as mean ± SD (*n* = 3, *P < 0.05). **C** Immunoblot analysis showed that the phosphorylation levels of JAK2 and STAT3 in cancer cells were down-regulated by matrine. **D** The ratio of p-JAK2/JAK2 and p-STAT3/STAT3 were obviously reduced in cancer cells. Data are presented as mean ± SD (*n* = 3, *P < 0.05). **E** After matrine treatment, the phosphorylation levels of PI3K and Akt were markedly decreased in cancer cells. **F** The bar graphs showed mean ± SD of the phosphorylation ratio of PI3K and Akt in cancer cells from three independent experiments (*P < 0.05).

Researches have showed that the MEK/ERK signaling pathway plays an important role in regulating cell proliferation, differentiation, and apoptosis, thus blocking it can inhibit the proliferation of tumor cells [12]. The expression and phosphorylation levels of MEK1/2 and ERK1/2 in cancer cells were detected by western blot to evaluate the effect of matrine on the MAPK/ERK signaling pathway. The results demonstrated that matrine decreased the phosphorylation levels of ERK1/2 and MEK1/2 in different cancer cells, without change of protein expression (Fig. 5A). The phosphorylation ratios of MEK1/2 and ERK1/2 were significantly reduced in HT-29, MCF7, A549, BxPC-3, SKOV3, and HeLa cells (Fig. 5B). Collectively, the phosphorylation levels of MAPK/ERK signaling pathway in cancer cells were dramatically inhibited by matrine.

The association between the JAK/STAT pathway and tumorigenesis is an important clue of tumor biology. STAT3 is an important transcription factor for gene transcription involved in cell proliferation, differentiation, apoptosis, and angiogenesis in a variety of cells [24]. Sustained activation of STAT3 occurs in many cancers, and can promote tumor growth, survival, and progression [25]. To investigate the effect of matrine on JAK2/STAT3 signaling pathway, the optimal concentrations of matrine were applied to treat cancer cells. Immunoblot analysis showed that the phosphorylation levels of JAK2 and STAT3 in cancer cells were down-regulated by matrine (Fig. 5C). The phosphorylation ratios of JAK2 were drastically decreased in HT-29, MCF7, and BxPC-3 cells, and the phosphorylation ratios of JAK2 and STAT3 were obviously reduced in HT-29, MCF7, A549, BxPC-3, SKOV3, and HeLa cells (Fig. 5D). Therefore, matrine could significantly suppress the phosphorylation levels of JAK2/STAT3 signaling pathway in cancer cells.

The PI3K/Akt signaling pathway is one of the essential pathways that regulate biological behaviors such as cell proliferation and invasion [26, 27]. Activation of PI3K/Akt pathway is often achieved by its phosphorylation, thus we examined the changes of the phosphorylation levels of PI3K and Akt in cancer cells with matrine treatment. As shown in Fig. 5E, after matrine treatment, the phosphorylation levels of PI3K and Akt in cancer cells were decreased. The phosphorylation ratios of PI3K and Akt were markedly decreased in HT-29, MCF7, A549, BxPC-3, SKOV3, and HeLa cells (Fig. 5F). The results above suggested that matrine significantly inhibited the activation of PI3K/Akt signaling pathway in cancer cells.

Meanwhile, IHC staining was performed to confirm that the phosphorylation levels of MEK1/2, ERK1/2, JAK2, STAT3, PI3K, and Akt in tumor tissues of mice inoculated with A549, BxPC-3, and SKOV3 cells, respectively. The results demonstrated that the phosphorylation levels of these proteins were significantly decreased in tumors with matrine treatment compared with the control group (Supplementary Fig. S4).

### Matrine regulates the proliferation related phosphorylation signaling pathways via targeting Src

**Fig. 6:**
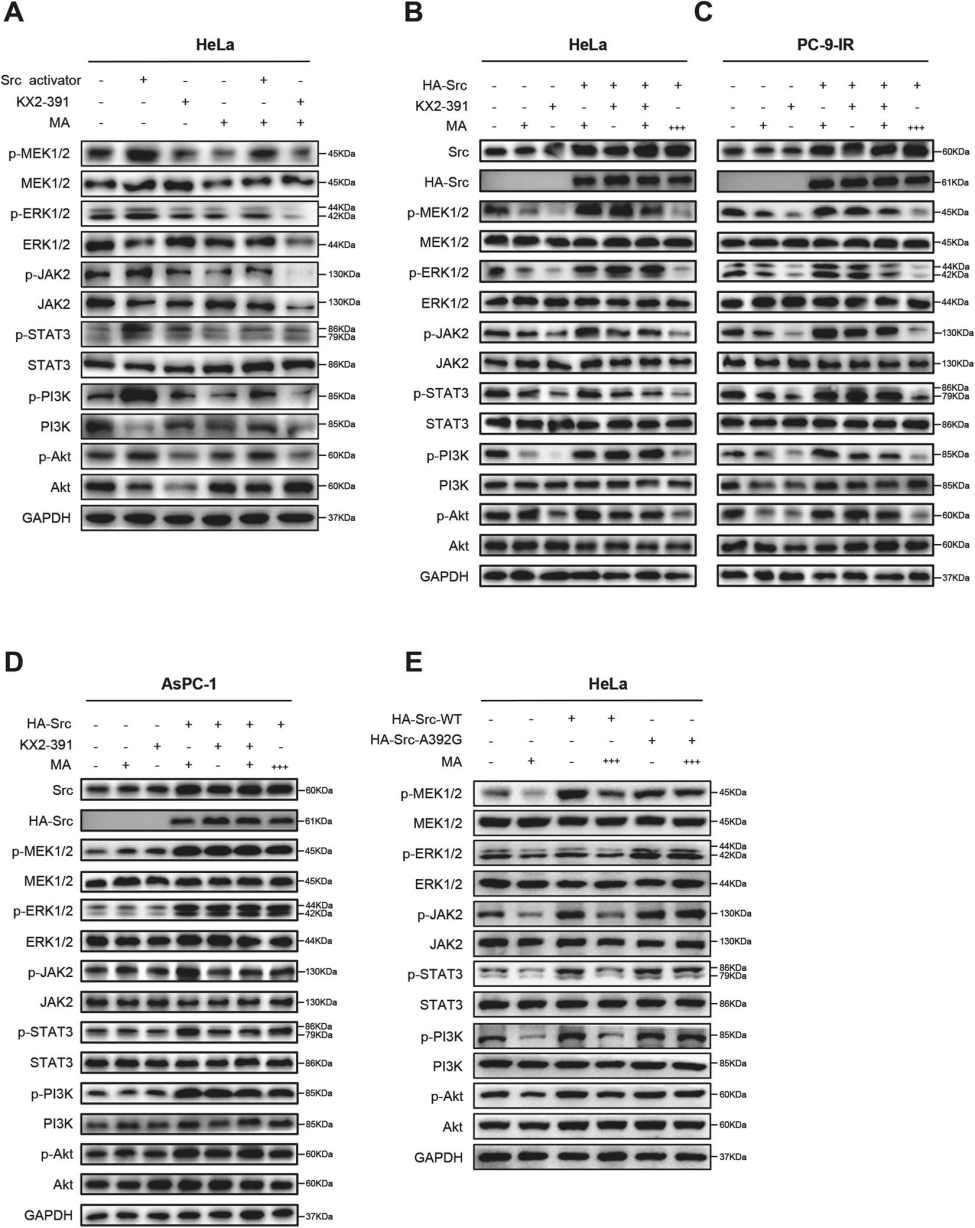
Involvement of Src in matrine-mediated phosphorylation signaling pathways. **A** HeLa cells were treated with Src activator (50 nM) or Src inhibitor KX2-391 (100 nM), and then exposed to matrine (2.5 mg/mL) for 24 h, respectively. The phosphorylation levels of MAPK/ERK, JAK2/STAT3, and PI3K/Akt signaling pathways were examined. GAPDH was used as a control. **B–D** Human cervical cancer cell HeLa, gefitinib-resistant human lung cancer cell PC-9-IR, and human pancreatic cancer cell AsPC-1 were transfected with plasmid pcDNA3.1-HA-Src, then treated with matrine, KX2-391(100 nM), and excessive matrine, respectively. The phosphorylation levels of MEK1/2, ERK1/2, JAK2, STAT3, PI3K, and Akt in HeLa (**B**), PC-9-IR (**C**), and AsPC-1 (**D**) were detected by western blot, respectively. GAPDH was used as a control. **E** Excessive matrine treatment could decrease the phosphorylation levels of various signaling proteins in HeLa cells overexpressing pcDNA3.1-HA-Src-WT, but could not do so in HeLa cells overexpressing pcDNA3.1-HA-Src-A392G (“+”: HeLa, 2.5 mg/mL; PC-9-IR, 2.5 mg/mL; AsPC-1, 3.0 mg/mL. “+++”: triple concentrations).

Protein phosphorylation is the most widespread class of PTMs in signal transduction [28]. Src interacts with several protein-tyrosine kinase receptors, such as EGFR, c-Met, PDGFR and IGFR, and then participates in pathways regulating cell survival, proliferation, and regulation of gene expression [29]. Once activated, Src acts as an upstream regulator of the Ras/MAPK and PI3K/Akt pathways inducing malignant transformation [30, 31]. To further verify that Src is the direct target of matrine in regulating the phosphorylation signaling pathways in cancer cells, Src activator peptide was delivered to cancer cells using a DirectX peptide transfection kit (Panomics). HT-29, MCF7, A549, BxPC-3, SKOV3, and HeLa cells were treated with Src activator or Src inhibitor KX2-391, and then exposed to matrine for 24 h at its optimal concentration, 2.5, 2.5, 3.0, 3.0, 2.0, and 2.5 mg/mL in HT-29, MCF7, A549, BxPC-3, SKOV3, and HeLa cells, respectively. The phosphorylation levels of MAPK/ERK, JAK2/STAT3, and PI3K/Akt signaling pathways in cancer cells were examined, respectively. The results demonstrated that Src activator peptide increased and KX2-391 decreased the phosphorylation levels of MEK1/2, ERK1/2, JAK2, STAT3, PI3K, and Akt in cancer cells. Their protein phosphorylation levels were further inhibited after exposed to matrine (Fig. 6A and Supplementary Fig. S5).

Subsequently, human cervical cancer cell HeLa, gefitinib-resistant human lung cancer cell PC-9-IR (mutant of EGFR, upstream protein of Src kinase), and human pancreatic cancer cell AsPC-1 (mutant of Ras, downstream protein of Src kinase) were transfected with plasmid pcDNA3.1-HA-Src, and then treated with matrine or KX2-391, respectively. The phosphorylation levels of MEK1/2, ERK1/2, JAK2, STAT3, PI3K, and Akt in cancer cells were detected by western blot. The results showed that both matrine and KX2-391 could inhibited the phosphorylation levels of these proteins in HeLa cells and EGFR mutant PC-9-IR cells. Consistent with our expectation, Src overexpression may rescue the phosphorylation levels of these proteins, and adding an excess amount of MA may decrease their phosphorylation levels again (Fig. 6B, C). Meanwhile, the protein phosphorylation levels of MEK1/2, ERK1/2, JAK2, STAT3, PI3K, and Akt had no significant change by matrine or KX2-391 in Ras mutant AsPC-1 cells, and Src overexpression, matrine, KX2-391, or excess amount of MA had no obvious effect on their phosphorylation levels (Fig. 6D).

To solidly prove that Src–matrine interaction plays a critical role in the regulation of cancer cell proliferation and phosphorylation signaling pathways, a mutant plasmid of pcDNA3.1-HA-SRC-A392G was constructed, which loses the binding ability to matrine without affecting normal kinase activity of Src (Fig. 3F and Supplementary Fig. S6). Transfected with plasmid pcDNA3.1-HA-Src-WT or pcDNA3.1-HA-Src-A392G, HT-29, MCF7, A549, BxPC-3, SKOV3, and HeLa cells were treated with excessive matrine, respectively. MTT assay and western blot results showed that excessive matrine treatment could obviously inhibit the cell proliferation and decrease the phosphorylation levels of various signaling proteins in cancer cells overexpressing pcDNA3.1-HA-SRC-WT, but failed to do so in cancer cells overexpressing pcDNA3.1-HA-SRC-A392G (Fig. 6E and Supplementary Figs. S7 and S8). These results indicated that Src–matrine interaction plays a vital role in the regulation of cell proliferation and phosphorylation signaling pathways. Collectively, all these data convincingly demonstrated that matrine inhibited the proliferation of cancer cells and related phosphorylation signaling pathways via targeting Src protein.

## Discussion

During the past few decades, unprecedented advances have been made in molecular biology and related technologies, which have greatly enhanced the understanding of pathogenesis of cancer, and then improved the therapy of cancer [32]. Chemotherapy and molecular targeted therapy are the main remedies to inhibit the growth and progression of cancer. Unfortunately, chemotherapeutic drugs showed significant toxicity to the normal cells. As a revolutionary therapy, molecular targeted agent may preferentially kill cancer cells by acting on specific target in them [33]. However, drug resistance is its Achilles’s hell because of the mutation of the targeted gene or protein, which causes the targeted agent to be futile within 6–12 months. To overcome the shortcomings of chemotherapeutic drugs and molecular targeted agents, it is urgent to develop novel anticancer drugs. With the advantages of wide spectrum of pharmacological activities and low side toxicity, the discovery and development of novel natural compounds has become a new hotspot in antitumor research.

Modern pharmacological studies have shown that Kushen has a long history of use for the treatment of tumors, inflammation, and other diseases in traditional Chinese medicine [34]. Matrine is an alkaloid isolated from *Sophora flavescens* Aiton, possessing a wide spectrum of biological activities [35]. Many researches have showed that matrine has antitumor effect on various types of cancers [36, 37]. However, its direct targets and molecular mechanisms have not been identified yet. The present study demonstrated that matrine inhibited the proliferation of cancer cells in vitro and in vivo. Meanwhile, matrine–amino coupling resins (MA beads) were prepared and used to pull down the targeted proteins of cancer cells.

Src is a non-receptor tyrosine kinase, and its overexpression or overactivation has been strongly implicated in the development, maintenance, and progression of human cancers, which indicates that Src may represent a compelling target for the cancer treatment [38, 39]. In our study, LC-MS/MS analysis identified that Src was the target protein of matrine. Furthermore, it was validated that Src was the direct target of matrine by CETSA and DARTS. Moreover, Src segmented plasmids were constructed and overexpressed in HEK-293 cells, pull-down experiment by matrine-amino coupling resins showed that matrine bound directly to the Src kinase domain. Molecular docking prediction and pull-down assay unveiled that a strong hydrogen-bonding interaction existed between matrine and the residue of Ala392 in Src kinase domain, and it participated in matrine–Src interaction. To date, ten additional kinases with homology to Src have been identified in human genome and collectively are referred to as the Src family kinases (SFKs). Intriguingly, although the proportion is very low comparing with Src protein, Lyn (tyrosine-protein kinase Lyn) was also been identified in the data of LC-MS/MS analysis as shown in Supplementary Table S1.

Studies have shown that Src can interact with several signaling transduction proteins related to proliferation, such as receptor tyrosine kinases, signal transducers and activators of transcription, G proteins, the mitogen-activated protein kinase ERK2. Src is the hub of several signaling pathways, including Ras/Raf/ERK1/2, PI3K/Akt, and STAT3, resulting in cell proliferation, survival, invasion, migration, and angiogenesis [40, 41]. In this study, the effect of matrine on Src kinase activity in cancer cells was examined. The results indicated that Src kinase activity was inhibited by matrine in a non-ATP-competitive manner, and its phosphorylation levels on tyrosine 419 were also decreased by matrine. Meanwhile, matrine down-regulated the phosphorylation levels of MAPK/ERK, JAK2/STAT3, and PI3K/Akt signaling pathways by targeting Src in cancer cells.

**Fig. 7:**
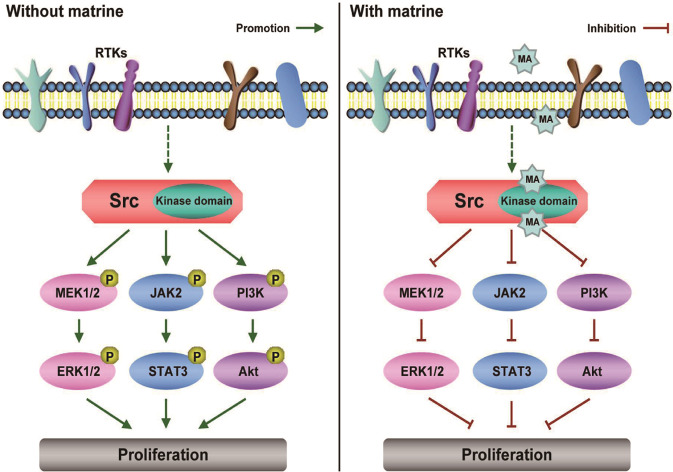
Schematic diagram of matrine inhibiting the proliferation of cancer cells. Src promotes the proliferation of cancer cells via the MAPK/ERK, JAK2/STAT3, and PI3K/Akt phosphorylation signaling pathways. Matrine may target Src protein and inhibit its kinase activity to inhibit the proliferation of cancer cells by blocking the related phosphorylation signaling pathways.

In summary, we first designed and synthesized matrine–amino coupling resins (MA beads), aiming to explore the direct targets of matrine. Our findings demonstrated that Src was the target of matrine in cancer cells, and Src kinase domain was the binding region of matrine. Matrine targeted Src, inhibited its kinase activity and tyrosine phosphorylation to inhibit the proliferation of cancer cells by down-regulating the downstream MAPK/ERK, JAK2/STAT3, and PI3K/Akt phosphorylation signaling pathways (Fig. 7). Therefore, matrine can be used as the leading compound for the development of active compounds of traditional Chinese medicine as novel targeted therapy drugs against cancer. Next step, chemically modifying the structure of matrine is necessary to enhance the specific binding with Src protein and decrease the effective dose of matrine. Meanwhile, the development of cancer comprises several aspects, such as proliferation, invasion, apoptosis, angiogenesis, etc. Further investigations are needed to explore whether other proteins act as the targets of matrine in the cancer cells in the future.

## Materials and methods

### Cell culture

Human colon cancer cell HT-29, human breast cancer cell MCF7, human non-small cell lung cancer cell A549, human pancreatic cancer cell BxPC-3, human ovarian cancer cell SKOV3, human cervical cancer cell HeLa, and human embryonic kidney cell HEK-293 were all obtained from the American Type Culture Collection (Manassas, VA, USA). Gefitinib-resistant human lung cancer cell PC-9-IR was obtained from Cobioer Biotechnology Co., Ltd (Nanjing, China) and they were identified by STR profiling without mycoplasma contamination. PC-9-IR cells were cultured in RPMI1640 medium containing additional 500 nM Gefitinib (Topscience, T1181) to maintain drug resistance. HT-29 cells were cultured in McCoy’s 5A medium, BxPC-3 and SKOV3 cells were grown in RPMI1640 medium, MCF7, A549, HeLa, and HEK-293 cells were maintained in Dulbecco’s modified Eagle’s medium (DMEM) supplemented with 10% fetal bovine serum (FBS) and 1% penicillin and streptomycin. All cells were incubated at 37 °C under a humidified 5% CO_2_ incubator.

### Reagents and antibodies

Matrine and Sophocarpine were purchased from Xi’an Natural Field Bio-Technique Co., Ltd (Xi’an, China). Matrine was dissolved in phosphate buffer saline (PBS) to a stock concentration of 100 mM. KX2-391 was purchased from Topscience Co., Ltd (Shanghai, China). Src activator peptide (sc-3052) was purchased from Santa Cruz Biotechnology (Santa Cruz, CA, USA).

Antibody against c-Src (sc-8056) was purchased from Santa Cruz Biotechnology. Antibody against HA (TA180128) was obtained from OriGene Technologies, Inc. (Maryland, USA). Antibodies against GST (#5475), phospho-ERK1/2 (T202/Y204) (#4370), ERK1/2 (#4695), phospho-MEK1/2 (S221) (#2338), MEK1/2 (#9122), phospho-JAK2 (Y1007/1008) (#3771), phospho-STAT3 (#9145), STAT3 (#4904), PI3K (#4292), phospho-Akt (S473) (#4060), Akt (#9272), β-actin (#4970), and GAPDH (#5174) were purchased from Cell Signaling Technology, Inc. (Beverly, MA, USA). Antibody against JAK2 (17670-1-AP) was obtained from Proteintech Group, Inc. (Chicago, USA). Antibody against phospho-PI3K (Y607) (ab182651) was purchased from Abcam (Cambridge, MA, USA). Phospho-Src (Tyr419) antibody (AF3162) was obtained from Affinity Biosciences Ltd (USA). Antibodies against Ki67 (MA5-14520) and PCNA (13-3900) were purchased from Thermo Fisher Scientific (MA, USA). HRP-conjugated anti-mouse and anti-rabbit secondary antibodies were obtained from Santa Cruz Biotechnology (Santa Cruz, CA, USA).

### MTT assay

The effect of matrine on the proliferation of cancer cells, including human colon cancer cell HT-29, human breast cancer cell MCF7, human non-small cell lung cancer cell A549, human pancreatic cancer cell BxPC-3, human ovarian cancer cell SKOV3, and human cervical cancer cell HeLa, were determined by MTT assay. Cells were exposed to matrine at various concentrations (0, 0.5, 1.0, 2.0, and 3.0 mg/mL) for the indicated time periods (0, 24, 48, and 72 h). The absorbance at 570 nm wavelength was measured using a microplate reader (Model 680, Bio-Rad, USA).

### Animal studies

BALB/c nude mice, aged 4–6 weeks, weighed 18–22 g, were obtained from the Medical Laboratory Animal Center of Tongji Medical College and maintained in pathogen-free conditions. This study was carried out in accordance with the guidelines approved by the Institutional Animal Care and Use Committee of Tongji Medical College, Huazhong University of Science and Technology, Wuhan, China.

To investigate the effect of matrine on tumor proliferation in vivo, the subcutaneous xenograft tumor models were established. BALB/c nude mice were subcutaneously injected with human non-small cell lung cancer cell A549, human pancreatic cancer cells BxPC-3, and human ovarian cancer cell SKOV3, respectively. Seven days after injection, mice were randomly divided into two groups, respectively (*n* = 4). The animals were treated with intraperitoneal injection of matrine or normal saline three times a week. Tumor size was measured by using a caliper and was calculated by using the following formula: volume = 1/2 × (length × width^2^). After inoculation, the mice were euthanized for assessing tumor load.

### 2-Amino-ethoxymatrine synthesis (P1)

Sophocarpine was dissolved in ethanolamine with sodium metal. The reaction was maintained at 60 °C for 30 h with gentle stirring. The product P1 was purified by column chromatography as a brown solid, yield: 65%. HRMS: calculated, C_17_H_29_N_3_O_2_ [M + H]^+^: 308.23, found 308.2337.

### Matrine-amino coupling resin synthesis (MA beads, P3)

2-Amino-ethoxymatrine was dissolved in coupling buffer. AminoLink^®^ coupling resin (Thermo Fisher Scientific, USA) and cyanoborohydride solution were added to the solution, and the reaction mixture was incubated by end-over-end rocking at 4 °C overnight. The resins were washed with wash solution and degassed buffer containing 0.05% sodium azide, and the matrine-amino coupling resins were stored at 4 °C.

### Affinity pull-down assay and MS analysis

Cell lysates were harvested and incubated with free amino coupling resins (control beads) or matrine–amino coupling resins (MA beads) at 4 °C with gentle rotation. To disturb the specific interaction between matrine–amino coupling resins and its target, free matrine (MA) was mixed with cell lysates before the binding experiment described above. The beads-bound proteins were separated by SDS-PAGE and visualized by Coomassie brilliant blue staining. The protein-containing band in the gel was excised, followed by in-gel digestion and analysis by LC-MS/MS (SpecAlly Life Technology Co., Ltd, Wuhan, China).

### Cellular thermal shift assay

Briefly, cell lysates were harvested and equally divided into two tubes for incubation for 3 h at room temperature with matrine or PBS. The lysates were aliquoted and each sample was heated at designated temperatures ranging from 42 to 64 °C for 3 min, followed by cooling for 3 min at room temperature. The soluble fractions from precipitates were separated by centrifuging at 13,000 r.p.m. for 25 min and then analyzed by western blot.

### Drug affinity responsive target stability

Cells were lysed in lysis buffer containing 200 mM Na_3_VO_4_, 1 M NaF, and protease inhibitor cocktail (Thermo, Rockford, IL, USA). The lysates were 1:10 diluted with TNC buffer [500 mM Tris·HCl (pH 8.0), 500 mM NaCl, 100 mM CaCl_2_] and equally divided into two aliquots, and then treated with matrine or PBS for 1 h at room temperature. After incubation, samples were digested with pronase (Roche Pharmaceutical Ltd, Switzerland) at room temperature for 30 min, and reactions were ceased by adding the loading buffer. Samples were loaded onto 10% SDS-PAGE gels and analyzed by western blot.

### Construction of segmented and mutant plasmids

The cDNA of Src was kindly provided by Dr. Jia-Huai Han (School of Life Sciences, Xiamen University, Xiamen, China). According to the structure and functions of Src, constructs containing SH4, Unique, and SH3 domain (USH3); SH4 and Unique domain (SH4-UD); SH3 domain; SH2 domain and Kinase domain encode amino acids 1–150, 1–85, 86–150, 151–269, and 270–536, respectively. Briefly, the genes of full-length Src and Src fragments (USH3, SH4-UD, SH3, SH2, and Kinase) were amplified and inserted into pcDNA3.1-HA between *Bam*HI and *Xho*I. The mutant plasmids pcDNA3.1-HA-Kinase-A392G and pcDNA3.1-HA-Src-A392G were constructed according to the instructions of Fast Site-Directed Mutagenesis Kit (Tiangen Biotech Co. Ltd., Beijing, China). The primers are described in Supplementary Table S2.

### Construction, expression, and purification of recombinant proteins

The genes of full-length Src and Src kinase domain were ligated into the *Bam*HI and *Not*I sites of pGEX-4T-2, respectively. The GST-Src and GST-Kinase fusion proteins were expressed in the *Escherichia coli* strain BL21, induced by isopropyl 1-thio-β-d-galactopyranoside (IPTG) at 22 °C for 3 h. The fusion proteins were purified from bacterial cell lysates by glutathione-agarose beads. The primers are shown in Supplementary Table S3.

### Molecular modeling

Molecular docking study was performed using the Discovery Studio 4.5. The crystal structure of Src kinase domain (1YOJ) was obtained from the Protein Data Bank. Matrine was treated with ligand preparation and minimization model to investigate spatial binding pattern of matrine and Src kinase domain.

### Kinase activity assay

The activity of Src kinase was measured according to the instructions of Src kinase activity detection kit (GENMED Scientifics Inc., USA). Cells with matrine treatment were lysed supplemented with protease inhibitor cocktail (Thermo Scientific, Rockford, IL, USA). The activity of Src kinase in cancer cells was quantified at a wavelength of 340 nm by a SpectraMax i3x multifunctional microplate reader (Molecular Devices Corporation, CA, USA).

### Western blot

Cell lysates were harvested and proteins obtained from cell lysates were equally loaded onto 10% SDS-PAGE gels, electrophoresed, and transferred to nitrocellulose membranes. Blocked with 5% nonfat milk, the membranes were incubated with primary antibodies overnight, probed with the appropriate secondary antibodies at room temperature. Detection was performed with an enhanced chemiluminescence detection kit (Thermo Scientific, USA) by a ChemiDoc MP Imager (Bio-Rad). Loading was normalized with GAPDH or β-actin.

### Immunohistochemistry assay

Tumors and tissues of heart, liver, spleen, lung, and kidney in mice were embedded in paraffin, cut into 4 μm sections, and either H&E stained or treated with corresponding antibodies for IHC evaluation.

### Statistical analysis

All data presented are the mean ± SD from at least three independent experiments. An unpaired Student’s *t*-test was used to evaluate the difference between two different treatments. The differences between multiple groups were analyzed by one-way analysis of variance (ANOVA). *P* < 0.05 was considered to be statistically significant.

## Supplementary information

Supplementary Figures and Tables

Supplementary Figure Legends

## Data Availability

The data presented in this study are available upon reasonable request from the corresponding authors.
