# Supplementary material for: Src acts as the target of matrine to inhibit the proliferation of cancer cells by regulating phosphorylation signaling pathways
Source: Cell Death Dis. 2021 Oct 12;12(10):931. doi: 10.1038/s41419-021-04221-6 (PMC8511016; doi:10.1038/s41419-021-04221-6)
Supplement: Supplementary file 1 — Supplementary Figures and Tables [file 41419_2021_4221_MOESM1_ESM.doc]

**Supplemental data**


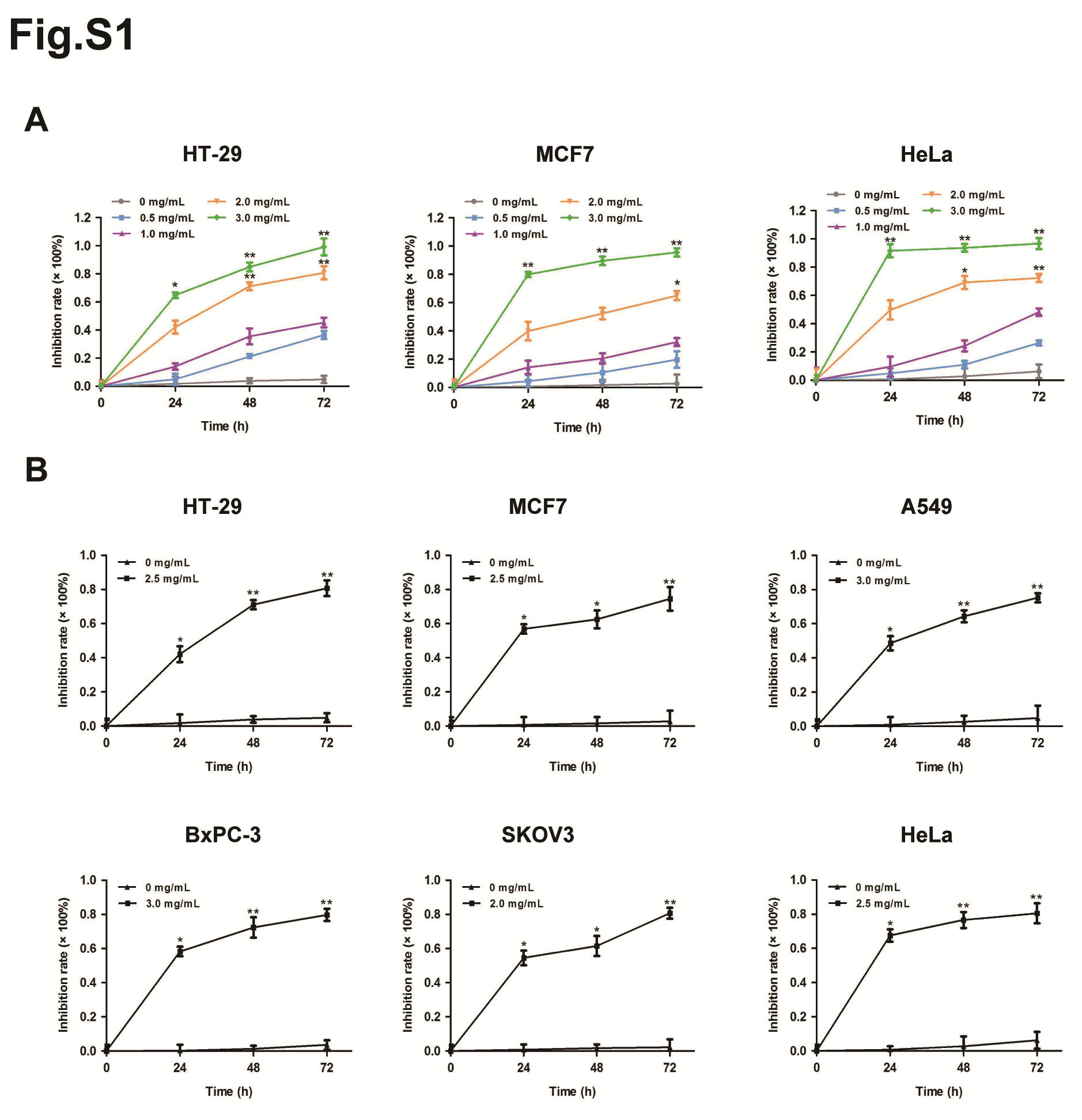


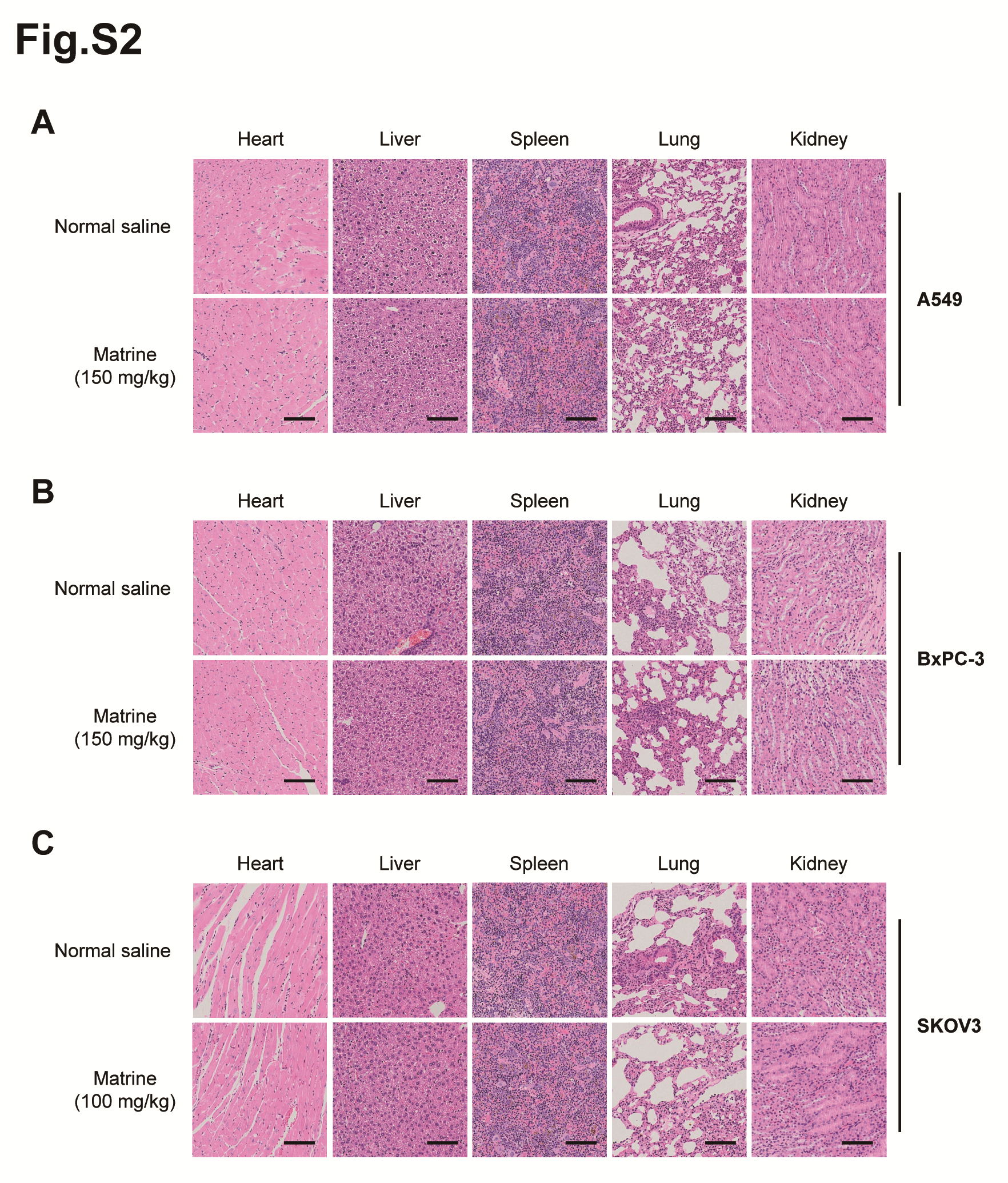


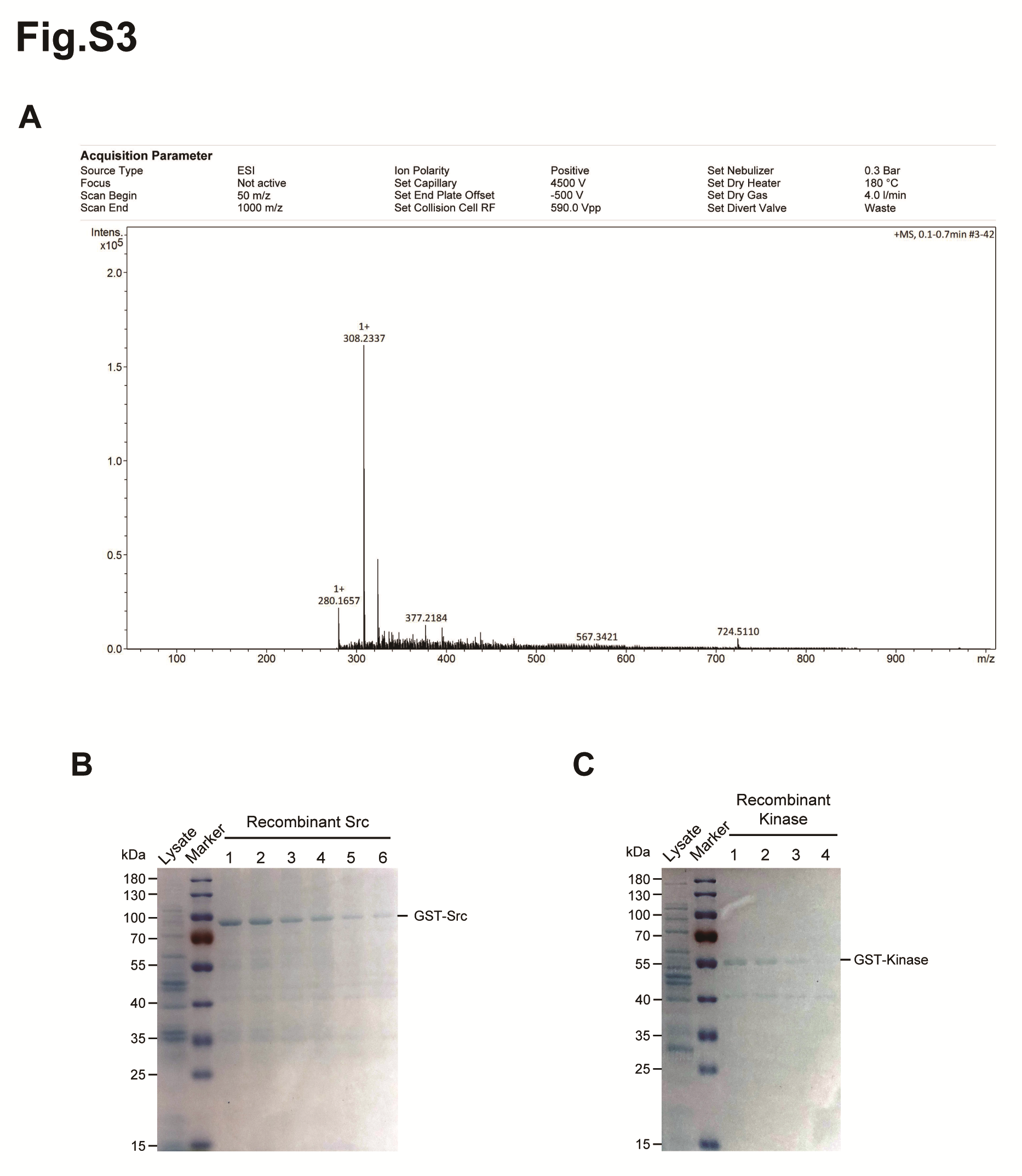


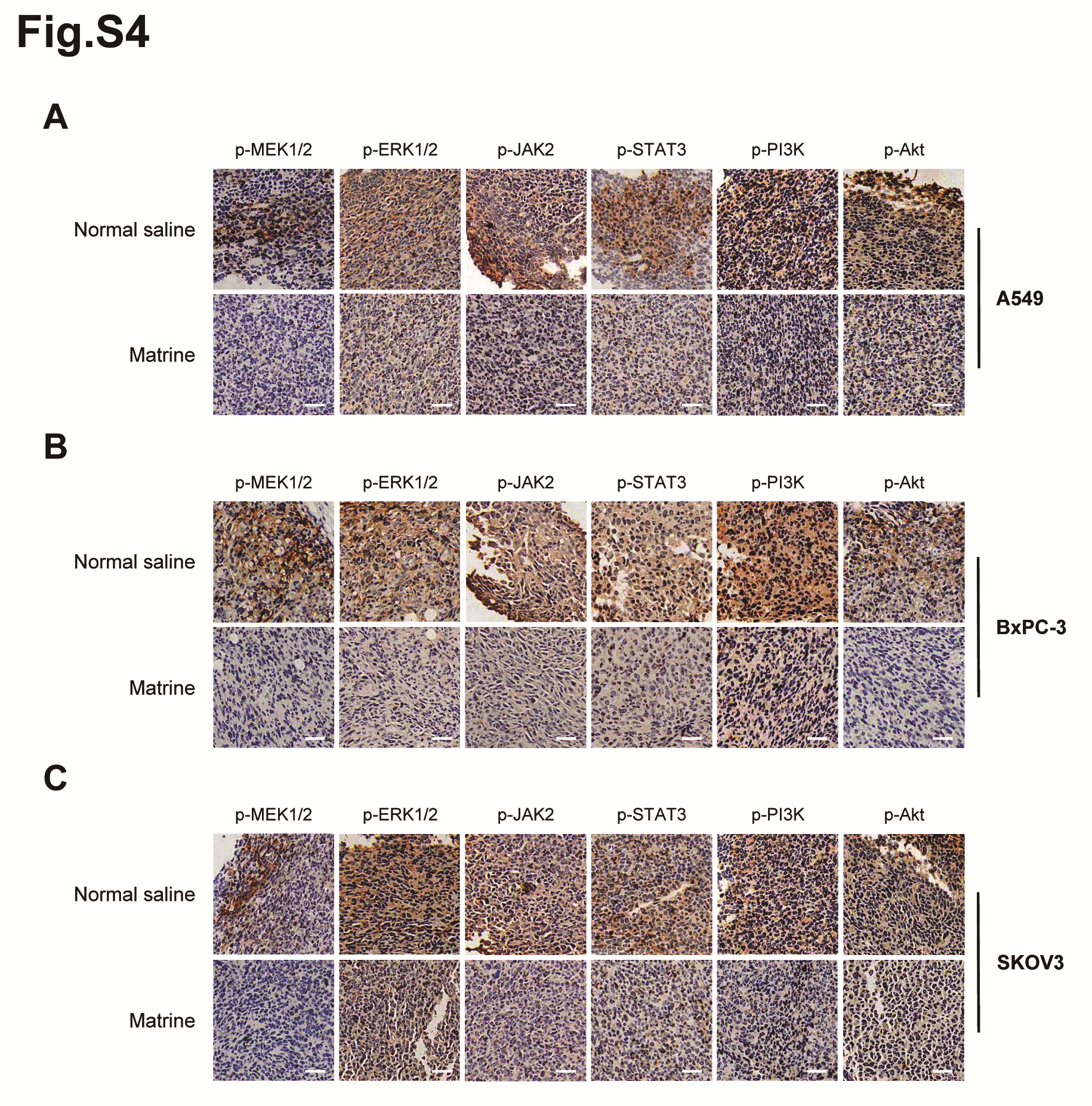


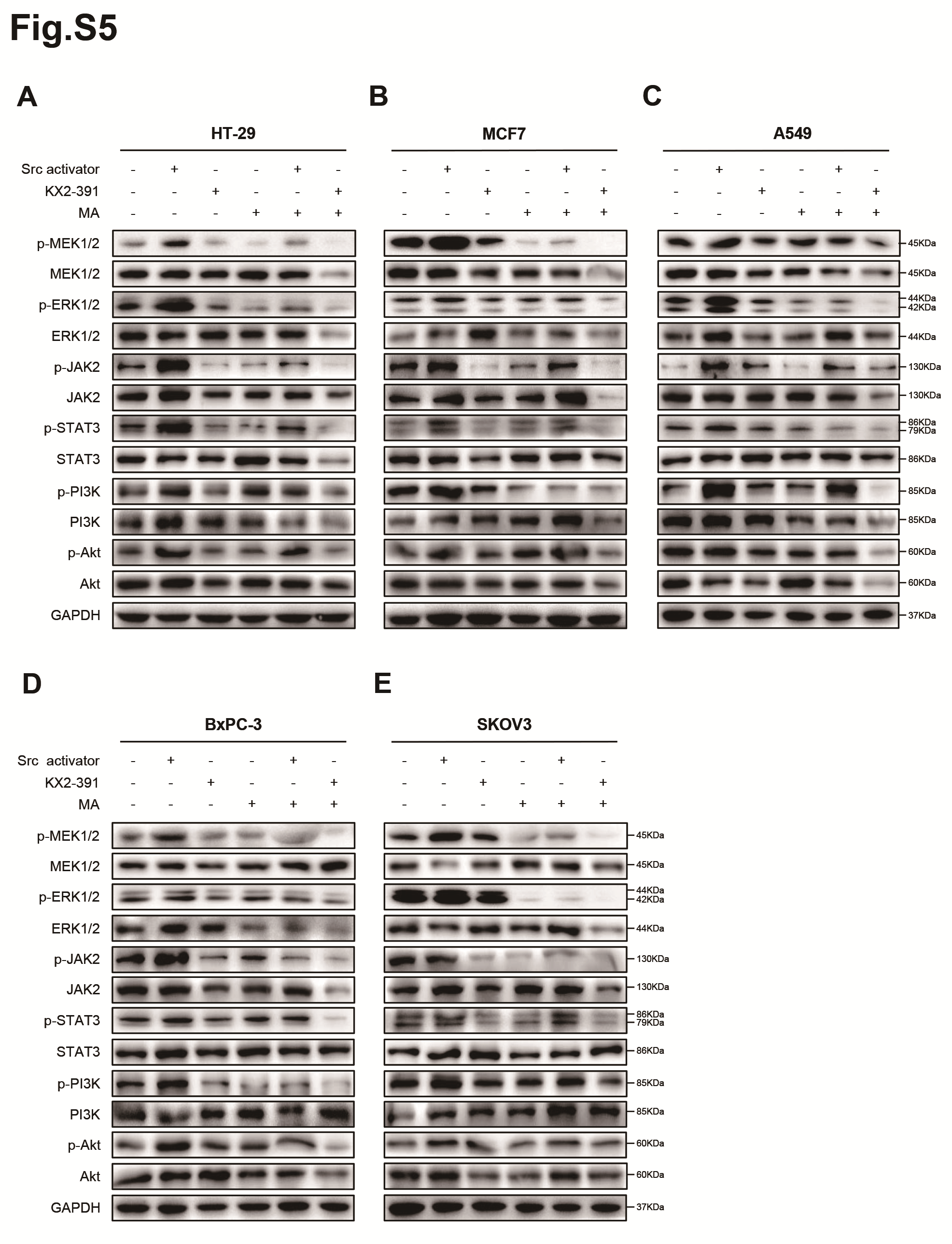


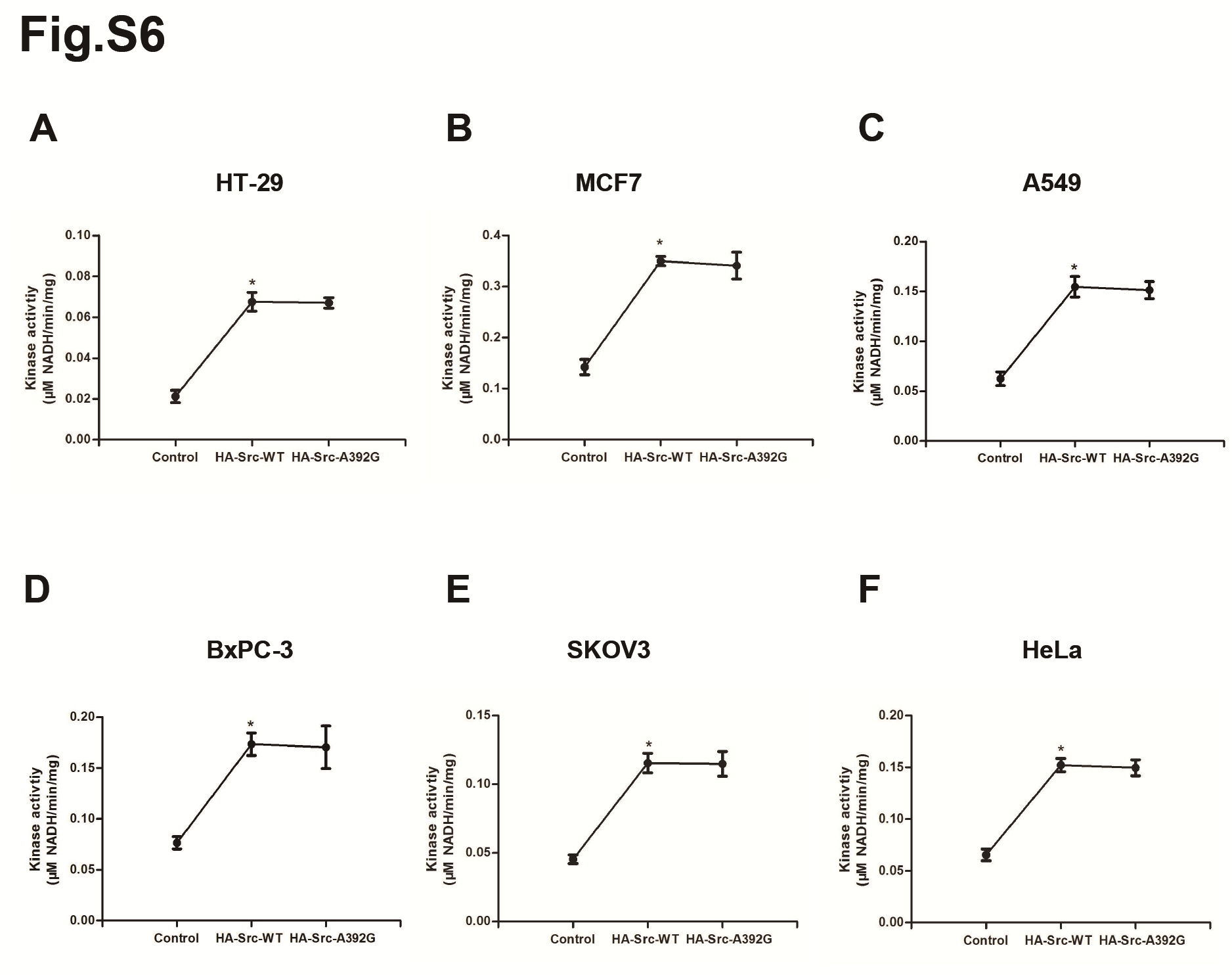


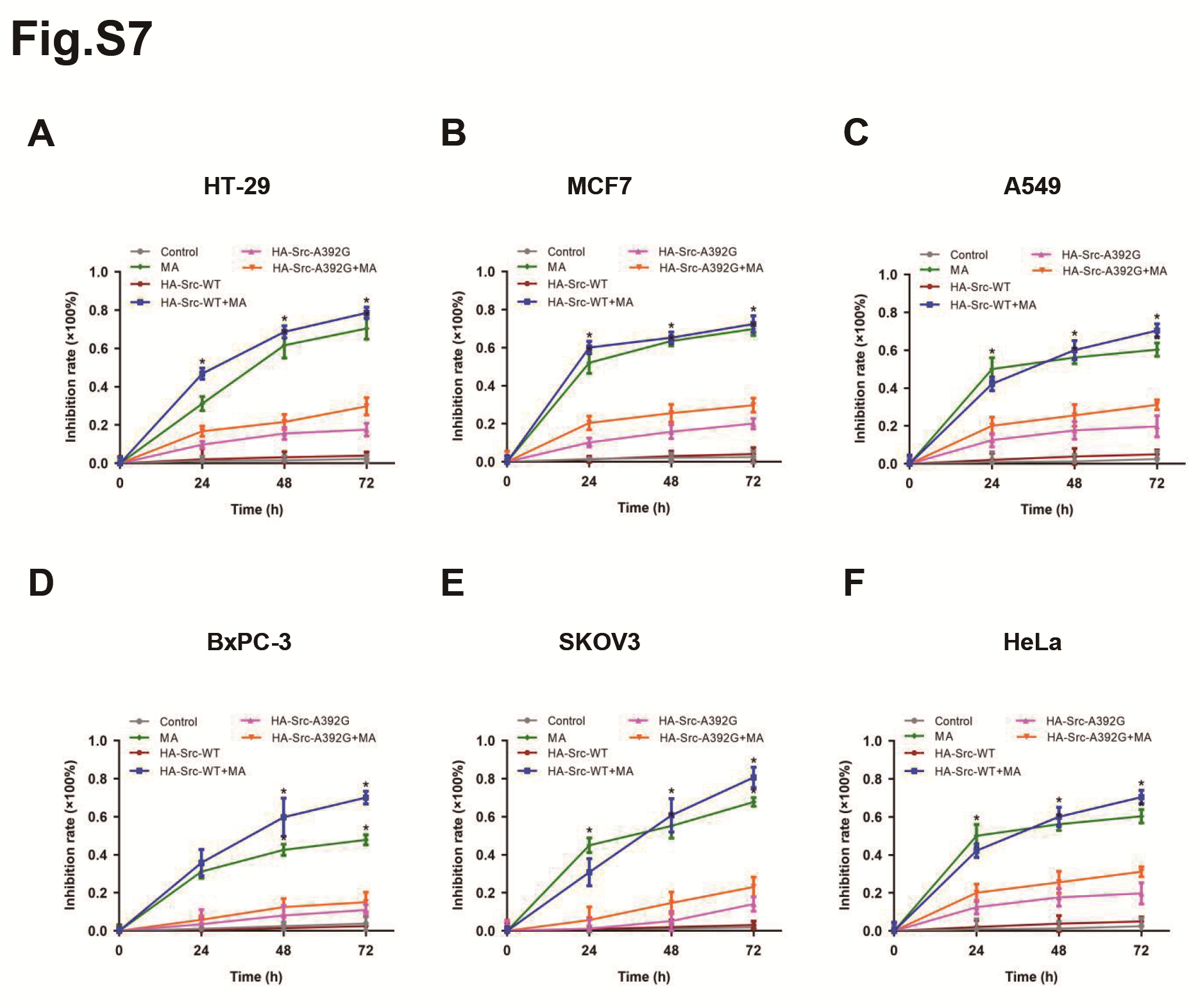


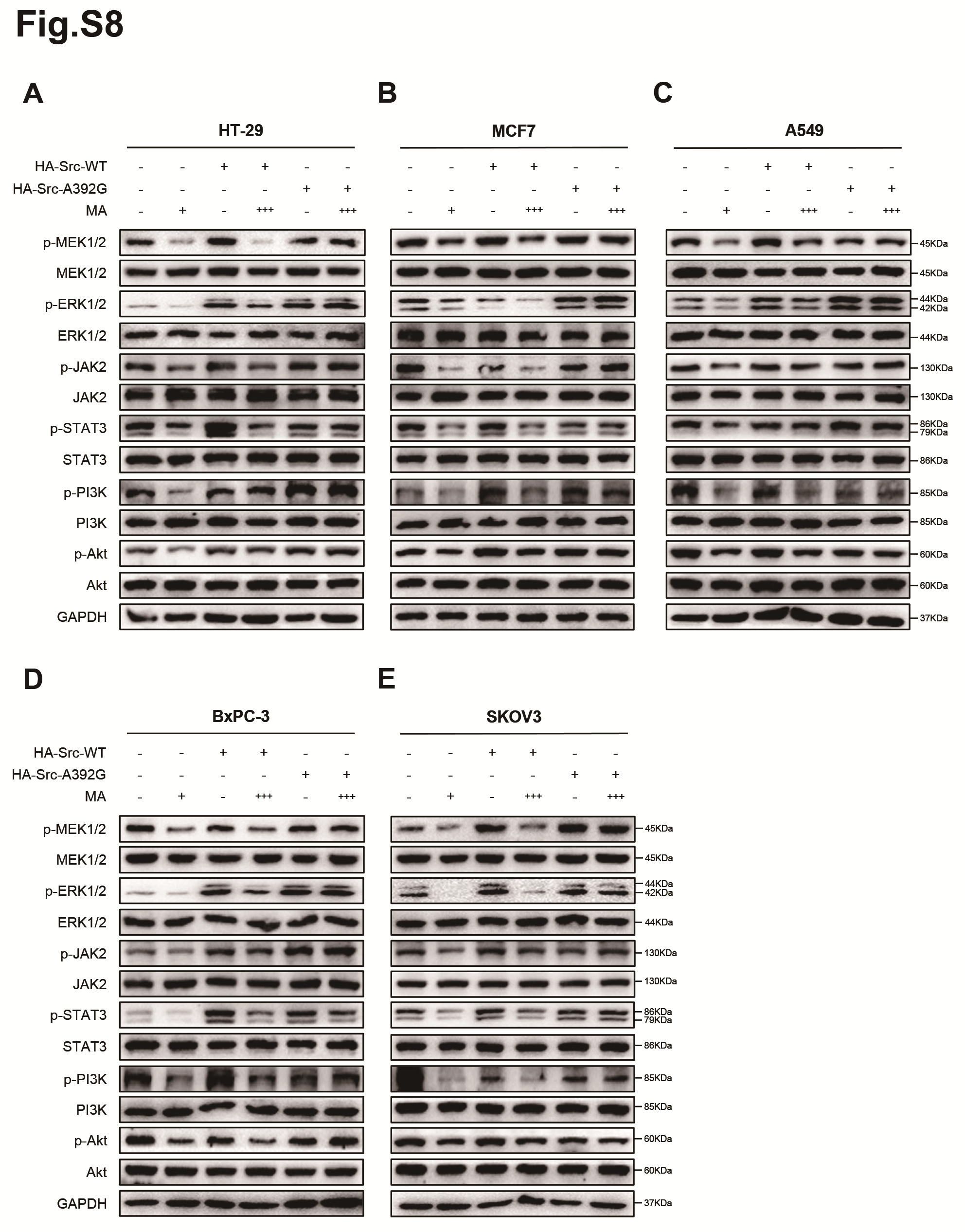


Table S1. The proteins identified by MS.

| N | Unused | % Cov (95) | Accession | Gene | Mw  (kD) | Equivalents | Others | Peptides  (95%) | Spectra |
| --- | --- | --- | --- | --- | --- | --- | --- | --- | --- |
| 1 | 13.37 | 12.69000024 | P12931 | SRC | 59.835 |  |  | 7 | 17 |
| 2 | 7.19 | 11.20000035 | P60709 | ACTB | 41.737 | P63261 | A0A2R8Y793;P62736;P63267;P68032;P68133 | 3 | 4 |
| 3 | 2.01 | 2.463000081 | P02768 | ALB | 69.367 | A0A087WWT3;A0A0C4DGB6;B7WNR0;C9JKR2;D6RHD5;H0YA55 |  | 1 | 1 |
| 4 | 1.93 | 2.140999958 | P11021 | HSPA5 | 72.333 |  |  | 1 | 1 |
| 5 | 1.85 | 0.7216 | P07948 | Lyn | 58.574 |  |  | 1 | 1 |
| 6 | 1.36 | 3.325999901 | P68363 | TUBA1B | 50.152 | Q71U36;Q9BQE3;A0A1W2PQM2;F5H5D3;F8VQQ4;F8VRK0;F8VRZ4;F8VS66;F8VVB9;F8VWV9;F8VX09 |  | 1 | 1 |

Table S2. Sequences of primers for plasmids.

| Genes | Primers |
| --- | --- |
| HA-Src | F: 5′-gttccagattacgctATGGGTAGCAACAAGAGCAA-3′ |
|  | R: 5′-cggccgcattctcgaCTAGAGGTTCTCCCCGGGCTG-3′ |
| HA-USH3 | F: 5′-gttccagattacgctATGGGTAGCAACAAGAGCAA-3′ |
|  | R: 5′-cggccgcattctcgaCTCCTCAGCCTGGATGGA-3′ |
| HA-SH4-UD | F: 5′-gttccagattacgctATGGGTAGCAACAAGAGCAA-3′ |
|  | R: 5′-cggccgcattctcgaTCCACCGGCCAGCGGGCC-3′ |
| HA-SH3 | F: 5′-gttccagattacgctGTGACCACCTTTGTGGCCCT-3′ |
|  | R: 5′-cggccgcattctcgaCTCCTCAGCCTGGATGGA-3′ |
| HA-SH2 | F: 5′-gttccagattacgctTGGTATTTTGGCAAGATCAC-3′ |
|  | R: 5′-cggccgcattctcgaCGACTCCCGAGGGATCTC-3′ |
| HA-Kinase | F: 5′-gttccagattacgctCTGCGGCTGGAGGTCAAGCT-3′ |
|  | R: 5′-cggccgcattctcgaCTAGAGGTTCTCCCCGGGCTG-3′ |
| HA-Kinase-A392G / HA-Src-A392G | F: 5′-CGTCCACCGGGACCTTCGTGGAGCCAACATCCTGGTGG-3′ |
|  | R: 5′-CCACGAAGGTCCCGGTGGACGTAGTTCATCCGCTCCAC-3′ |

Table S3. Primers used for recombinant proteins.

| Genes | Primers |
| --- | --- |
| GST-Src | F: 5′-atctggttccgcgtggatccATGGGTAGCAACAAGAGC-3′ |
|  | R: 5′-agtcacgatgcggccgcCTAGAGGTTCTCCCCGGGC-3′ |
| GST-Kinase | F: 5′-atctggttccgcgtggatccCTGCGGCTGGAGGTCAAG-3′ |
|  | R: 5′-agtcacgatgcggccgcCTAGAGGTTCTCCCCGGGCTG-3′ |
