# Supplementary material for: Src acts as the target of matrine to inhibit the proliferation of cancer cells by regulating phosphorylation signaling pathways
Source: Cell Death Dis. 2021 Oct 12;12(10):931. doi: 10.1038/s41419-021-04221-6 (PMC8511016; doi:10.1038/s41419-021-04221-6)
Supplement: Supplementary file 2 — Supplementary Figure Legends [file 41419_2021_4221_MOESM2_ESM.doc]

**Supplemental data**

**Figure S1.** **Matrine significantly inhibited the proliferation of cancer cells at the optimal concentrations.** (A) Human colon cancer cell HT-29, human breast cancer cell MCF7 and human cervical cancer cell HeLa were treated with matrine at concentrations of 0, 0.5, 1.0, 2.0 and 3.0 mg/mL for 0, 24, 48 and 72 h, respectively. MTT assay was used to examine the effect of matrine on the proliferation of cancer cells. Data are presented as mean ± SD (**P* < 0.05, ***P* < 0.01). (B) MTT assay was performed to determine the optimal concentrations of matrine in six types of cancer cells, including HT-29, MCF7, A549, BxPC-3, SKOV3 and HeLa. Data are presented as mean ± SD (**P* < 0.05, ***P* < 0.01). Results are representative of three experiments.

**Figure S2. Matrine had no obvious toxicity in vivo.** Xenograft models were established by subcutaneous injection of A549 (A), BxPC-3 (B) and SKOV3 (C) cells in BALB/c nude mice, respectively. H&E staining of heart, liver, spleen, lung and kidney tissues in mice with matrine or normal saline treatment (150 mg/kg in A549 and BxPC-3 cells, 100 mg/kg in SKOV3 cells, ip). Scale bar: 100 μm.

**Figure S3.** **Identification of 13α-(2-amino) ethoxymatrine and purification of recombinant proteins.** (A) The high-resolution mass spectrum (HRMS) of 13α-(2-amino) ethoxymatrine (P1). HRMS (ESI): calculated for [C_17_H_29_N_3_O_2_]^+^: 308.23, found 308.2337. High-resolution mass spectrum (HRMS) was gathered on a Bruker MicroTOF-Q III LC-MS instrument operating in electrospray ionization (ESI). (B,C) Expression and purification of the fusion proteins GST-Src (B) and GST-Kinase (C) and imaging with SDS-PAGE by Coomassie brilliant blue staining.

**Figure S4. Matrine inhibited the phosphorylation levels of proteins in tumors.** Xenograft models were established by subcutaneous injection of A549 (A), BxPC-3 (B) and SKOV3 (C) cells in BALB/c nude mice, respectively. IHC staining demonstrated a decrease in the phosphorylation levels of MEK1/2, ERK1/2, JAK2, STAT3, PI3K and Akt in xenografts of treated with matrine compared with those in control group. Scale bar: 50 μm.

**Figure S5. Src invovled in matrine-mediated phosphorylation signaling pathways.** HT-29, MCF7, A549, BxPC-3, SKOV3 cells were treated with Src activator (50 nM) or Src inhibitor KX2-391 (100 nM), then exposed to matrine at its optimal concentrations for 24 h, respectively. The phosphorylation levels of MAPK/ERK, JAK2/STAT3 and PI3K/Akt signaling pathways in HT-29 (A), MCF7 (B), A549 (C), BxPC-3 (D), SKOV3 (E) cells were examined, respectively. GAPDH was used as control (“+”: 2.5, 2.5, 3.0, 3.0, 2.0 mg/mL for HT-29, MCF7, A549, BxPC-3, SKOV3 cells, respectively).

**Figure S6. HA-Src-A392G retained the same kinase activity as HA-Src-WT.** The effects of HA-Src-WT or HA-Src-A392G on Src kinase activity in HT-29 (A), MCF7 (B), A549 (C), BxPC-3 (D), SKOV3 (E) and HeLa (F) cells were determined according to the instructions of Src kinase activity detection kit. Results are representative of three experiments (**P* < 0.05, ***P* < 0.01).

**Figure S7. Matrine treatment could not suppress the proliferation of cancer cells overexpressing pcDNA3.1-HA-Src-A392G.** HT-29 (A), MCF7 (B), A549 (C), BxPC-3 (D), SKOV3 (E) and HeLa (F) cells were transfected with plasmid pcDNA3.1-HA-Src-WT or pcDNA3.1-HA-Src-A392G, then treated with excessive matrine for 0, 24, 48 and 72 h, respectively. MTT assays were conducted to examine the Src-matrine interaction in the regulation of cell proliferation. Results are representative of three experiments (**P* < 0.05). (excessive matrine, 7.5, 7.5, 9.0, 9.0, 6.0, and 7.5 mg/mL matrine for HT-29, MCF7, A549, BxPC-3, SKOV3, and HeLa, respectively.)

**Figure S8. Matrine treatment could not suppress the phosphorylation levels of various signaling proteins in cancer cells overexpressing pcDNA3.1-HA-Src-A392G.** HT-29, MCF7, A549, BxPC-3, SKOV3 cells were transfected with plasmid pcDNA3.1-HA-Src-WT or pcDNA3.1-HA-Src-A392G, then treated with excessive matrine for 24h, respectively. The phosphorylation levels of MAPK/ERK, JAK2/STAT3 and PI3K/Akt signaling pathways in HT-29 (A), MCF7 (B), A549 (C), BxPC-3 (D), SKOV3 (E) cells were examined, respectively. GAPDH was used as control (“+”: 2.5, 2.5, 3.0, 3.0, 2.0 mg/mL for HT-29, MCF7, A549, BxPC-3, SKOV3 cells, respectively. “+++”: triple concentrations).

**Table S1. The proteins identified by MS.**

**Table S2. Sequences of primers for plasmids.**

**Table S3. Primers used for recombinant proteins.**
